# Childhood stunting and the metabolic syndrome components in young adults from a Brazilian birth cohort study

**DOI:** 10.1038/ejcn.2015.220

**Published:** 2016-01-06

**Authors:** L P Grillo, D P Gigante, B L Horta, F C F de Barros

**Affiliations:** 1 Departament of Nutrition, Vale of Itajaí University, Itajaí, Santa Catarina, Brazil; 2 Epidemiological Research Center, Epidemiology Postgraduate Program, Federal University of Pelotas, Rio Grande do Sul, Brazil; 3 Departament of Nutrition, Federal University of Pelotas, Rio Grande do Sul, Brazil

## Abstract

**Background/Objectives:**

The aim of this study was to investigate the association between stunting in the second year of life and metabolic syndrome components in early adulthood among subjects who have been prospectively followed-up since birth, in a city in Southern Brazil.

**Subjects/Methods:**

In 1984, we attempted to follow-up the entire cohort; the subjects were examined and their mothers interviewed. Stunting was defined by a length-for-age *Z*-score 2 s.d. or more below the mean, in accordance with the World Health Organization reference. Between 2004 and 2005, we again tried to follow the entire cohort; during this period the subjects were evaluated for the following metabolic syndrome components: high-density lipoprotein (HDL) cholesterol, triglycerides, random blood glucose, waist circumference and systolic and diastolic blood pressure. Family income at the time of the baby's birth, asset index, mother's education, mother's smoking during pregnancy and duration of breastfeeding were considered possible confounders. Linear regression was used in the unadjusted and adjusted analyses.

**Results:**

Among men, stunting was inversely associated with triglycerides (*β*=−11.90, confidence interval (CI)=−22.33 to −1.48) and waist circumference (*β*=−4.29, CI=−5.62 to −2.97), whereas for women stunting was negatively related to HDL-cholesterol (*β*=−4.50, CI=−6.47 to −2.52), triglycerides (*β*=−9.61, CI=−17.66 to −1.56) and waist circumference (*β*=−1.14, CI=−4.22 to −1.02). However, after controlling for confounding variables, these associations vanished.

**Conclusions:**

The findings suggest that stunting in childhood is not associated with metabolic syndrome components in young adults.

## Introduction

Stunting is the most prevalent form of malnutrition worldwide and is characterised by linear growth deficiency in childhood.^1^ In 2011, globally, 26% of children aged <5 years were stunted; in Latin America and the Caribbean there was a prevalence of 13%.^2^ In Brazil, stunting declined from 37% in 1974–1975 to 7% in 2006–2007^3^ and population-based birth cohort studies in the city of Pelotas (Brazil) in 1982, 1993 and 2004 stunting among 2-year-olds declined from 13.9 to 5.0%.^4^

Childhood malnutrition has been reported to increase the risk of obesity later in life.^5, 6, 7, 8^ Some studies have observed that malnutrition is associated with the following: morbidity and mortality; reduced physical; neurodevelopmental and economic capacity; elevated risk of metabolic disease in adulthood; and particularly with increased abdominal fat.^1, 9, 10, 11^ Furthermore, Walker *et al.*^12^ reported that abdominal obesity was higher among children with short stature, decreased body mass index and body fat in comparison with those with average stature.

Metabolic syndrome is a complex disorder associated with several cardiovascular risk factors including hypertension, increased triglycerides, decreased high-density lipoprotein (HDL)-cholesterol and glucose tolerance. These conditions are usually associated with central body fat deposition and insulin resistance.^11, 13^ In addition, epidemiological studies have indicated that progressive weight gain is an independent risk factor for developing metabolic syndrome and that central body fat deposition has a major role in some cardiovascular conditions.^14, 15, 16, 17^

To the best of our knowledge, no previous studies have examined the association between short stature in childhood and metabolic syndrome in adulthood. In the present study, our aim was to assess the relationship between the metabolic syndrome components and childhood stunting in young adults who have been followed-up since birth.

## Subjects and methods

In 1982, the maternity hospitals located in Pelotas, a city in Southern Brazil, were visited daily and the births identified. There were 5914 live births in families living in the urban area of the city; both babies and their mothers were examined, and their mothers were interviewed. These subjects have been followed-up several times. In 1984, a census was conducted in the urban area of the city, in search of the subjects belonging to the cohort. This led to 4934 children being examined, and considering the 227 known to have died, it resulted in a follow-up rate of 87.2%. Between 2004 and 2005, a new census was conducted in the urban area of Pelotas to identify the cohort members (mean age: 22.8 years). A total of 4297 cohort participants were interviewed (2213 men and 2084 women), resulting in a monitoring rate of 77.4%, after accounting for the 282 deaths identified until the period of data collection. Furthermore, 3832 subjects donated a blood sample. Further details about monitoring the subjects included in the Pelotas 1982 birth cohort study have been published elsewhere.^18, 19^

In the 1984 visit, the children were weighed using portable mechanical scales (CMS Weighing Equipment Ltd, London, UK) with 100 g accuracy, and their length was measured in the supine position using a stadiometer locally manufactured according to international specifications (AHRTAG; Healthlink Worldwide, London, UK). For this analysis, height-for-age was assessed according to the latest recommendations of the World Health Organization (WHO). Stunting was defined by a length-for-age *Z*-score 2 s.d. or more below the mean, in accordance with the WHO growth chart.^20^

The following metabolic syndrome components were assessed in the 2004–2005 survey, according to the NCEP-ATP III:^21^

- HDL-cholesterol was measured by using an ultrasensitive direct method, with a Selectra 2 analyser.
- Triglyceride was assessed by means of a colourimetric enzymatic method.
- Random blood glucose was assessed from fingertip blood by using a portable glucose meter (Accu-Chek Advantage, Roche, Mannheim, Germany).
- Waist circumference was measured in the narrowest part of the trunk, at the midpoint between the rib cage and the iliac crest, by using a flexible non-elastic tape applied over bare skin with cutoff points >102 cm and >88 cm for men and for women, respectively.
- Blood pressure was measured at the beginning and at the end of the interview using a digital wrist blood pressure monitor (Omron HEM-629, Kyoto, Japan). The mean values were used in the analyses. The mean arterial pressure was determined from systolic blood pressure and diastolic blood pressure values using the following formula: (MAP=SBP + (DBP × 2)/3).
- Body mass index was assessed as weight (in kilograms) divided by the square of height (in metres).

As regards quality control, scales were calibrated regularly, and 5% of the interviews were repeated; anthropometry standardisation sessions were carried out; the data were entered twice and the data set compared.

In this analysis, the following variables were considered possible confounders: family income at the time of baby's birth in minimum wages, household asset index (estimated using factor analysis and based on the ownership of household goods), maternal schooling in years, baby's birth weight, breastfeeding duration in months, maternal smoking habits during pregnancy, mother's height, pre-gestational body mass index and mother's skin colour.

Data analysis was performed using STATA software version 10.0 (College Station, TX, USA). Triglyceride levels were natural-log transformed because of the asymmetry of distribution, and the geometric mean is presented for this variable. In the bivariate analysis, the *χ*^2^-test was used. Means were compared using the *t*-test and analysis of variance. Adjusted analyses were carried out using linear regression analysis. Comparisons between categories were based on heterogeneity and linear trend tests for ordinal variables, and the lowest *P*-value was presented. Analyses were stratified according to gender, and if the *P*-value for interaction was <0.10, we considered this evidence of interaction. A significance level of 5% was used for all analyses.

This study was conducted according to the guidelines established in the Declaration of Helsinki, and all procedures involving human subjects were approved by the Ethics Committee of the Faculty of Medicine at the Federal University of Pelotas. Verbal informed consent was obtained from the caregivers in early phases of the study, and written informed consent was obtained from the cohort members in recent phases.

## Results

In 2004–2005, we evaluated 4297 subjects, collected blood samples from 3824 and evaluated random blood glucose of 3732 subjects. Differences in fasting time were controlled when these values were included in the multivariate analysis, thus reducing the possibility of differential information bias.

Table 1 shows the sample characteristics according to gender. Approximately 75% of the subjects self-reported as white; 41% of the mothers achieved from 5 to 8 years of schooling; 47% had a family income between one and three minimum wages; 23% of babies were breastfed for <6 months; and 35% of the mothers smoked during pregnancy. Most of the mothers (70%) exhibited normal weight when the pregnancy began, and two-thirds of them gained up to 13.7 kg during gestation. Almost a third of the cohort presented excessive weight (overweight or obesity) at the age of 23 years.

Table 2 shows the distribution of metabolic syndrome and its components, according to the nutritional status of individuals whose blood samples were collected. Prevalence of metabolic syndrome was 6.8% (*N*=264). As regards metabolic syndrome components, individuals stunting showed significantly higher prevalence of altered HDL-cholesterol; and normal individuals showed significantly higher prevalence of high triglycerides and central obesity. Individuals who do not have blood samples collected (*N*=465) showed similar prevalence with regard to the variables: mean arterial pressure, central obesity, overweight and obesity; but the values were not statistically significant (data not shown).

Table 3 shows the association between height-for-age *Z*-score at the age of 2 years and metabolic syndrome components assessed at the age of 23 years in men and women. Among men, stunting was associated with lower mean triglycerides levels (*P*<0.081) and waist circumference (*P*<0.0001). In women, the association was in the same direction for three components: HDL-cholesterol levels (*P*<0.0001); triglycerides levels (*P*=0.0076); and waist circumference (*P*=0.0055).

Table 4 shows the results of unadjusted and adjusted analyses of the association between stunting at the age of 2 years and metabolic syndrome components for both men and women. Among men, stunting was inversely associated with triglycerides (*β*=−11.90, confidence interval (CI)=−22.33 to −1.48) and waist circumference (*β*=−4.29, CI=−5.62 to −2.97), whereas for women *Z*-score increase was negatively related to HDL-cholesterol (*β*=−4.50, CI=−6.47 to −2.52), triglycerides (*β*=−9.61, CI=−17.66 to −1.56) and waist circumference (*β*=−1.14, CI=−4.22 to −1.02). On the other hand, after controlling for confounding variables these associations vanished. Further analyses using body mass index were also performed, but the results were similar (data not shown).

## Discussion

Conversely to the evidence from high-income countries indicating that nutritional deficits in childhood may program the development of chronic health conditions^22^ in a middle-income country such as Brazil, where the present study was conducted, the effects of nutritional condition at an early age were due to confounding by socioeconomic and maternal conditions.

However, a previous study from our research group revealed that men who were stunted at the age of 2 years and had abdominal obesity at the age of 23 years had higher C-reactive protein levels, suggesting an increased risk for chronic diseases.^23^ The correlation between stunting and altered glucose levels in childhood remains unknown. However, poor countries with accelerated urbanisation processes are known to be particularly vulnerable to the increase in prevalence of Type 2 diabetes.^24^ This association would be due to accelerated weight gain among subjects who had been previously undernourished.

The prospective design of our study made it less susceptible to information bias because variables were measured at the age of 2 years. Moreover, the low attrition rate decreased the chance of selection bias. On the other hand, the main limitation of the present study was related to variations in the duration of fasting before blood collection, considering that the fasting duration was lower than the recommended 12 h for most respondents. However, because data on fasting duration were collected, this variable was adjusted in the analyses. Another limitation was the absence of the burden and antecedents of hereditary chronic diseases in parents or siblings.

Nutritional stunting is caused by a combination of several factors including intrauterine and maternal malnutrition, inadequate quality or quantity of complementary foods ingested in childhood and poor nutrient absorption caused by a high prevalence of infection.^22^ Increasing evidence suggests a correlation between stunting in childhood and impairments in educational and economic performance in adulthood.^25, 26^

Prospective birth cohort studies conducted in Switzerland,^27^ the UK,^28^ China^29^ and low- and middle-income countries, including Brazil,^30^ Guatemala, India, the Philippines and South Africa,^16, 17^ suggest that rapid gain in body weight in mid-childhood increases the risk of chronic disease in adulthood.^31^ In addition, stunting in early life has been associated with high prevalence of hypertension in children^32, 33^ and adolescents.^34^ Therefore, recognising stunting in childhood as a risk factor for the changes in metabolic syndrome components in adulthood continues to be a contradictory topic. Our research group previously conducted another study of three prospective birth cohorts in the same city over a period of more than two decades that described secular trends in stunting at different ages, according to socioeconomic position. Between the 1982 and 2004 cohorts, stunting among 2-year-olds declined (from 13.9 to 5.0%), and in every visit the prevalence of stunting was inversely related to income.^4^

Cross-sectional studies conducted in Northeastern Brazil,^34^ involving 416 slum-dwelling adult subjects, revealed that systolic and diastolic blood pressure were inversely related to stature and were more prevalent in obese and short women (50%) than in obese and normal stature women. Another study^35^ investigated whether the health conditions of short mothers differed from those of normal stature mothers and revealed that short stature was independently associated with obesity, abdominal fat accumulation and blood pressure increase. Moreover, a recent study noted that short maternal stature was associated with low birth weight and short stature in children.^36^ A Finnish cohort study noted that pre- and post-natal growth patterns influence blood pressure and obesity in adults. Moreover, the rapid growth in post-natal infant stature was shown to be inversely correlated with systolic and diastolic blood pressure.^37^

Notably, data from the same cohort study as those of the present study^38^ suggested that malnutrition in childhood was not a risk factor for overweight and obesity in adolescents. In addition, stunting in children in Jamaica was not associated with serum levels of glucose, cholesterol and triglycerides in adolescence;^39^ however, adolescents with a short stature had abdominal obesity. Head *et al.*^40^ found no correlation between malnutrition at different ages and cholesterol levels in adulthood.

Another study involving obese and short-statured adult women investigated the association between abdominal obesity and insulin resistance.^34^ The results indicated a tendency towards an increase in insulin resistance. Moreover, multivariate logistic regression analysis found that stature was the primary factor associated with insulin resistance. Furthermore, short-statured women exhibited higher levels of glycated haemoglobin and low-density lipoprotein cholesterol and significantly lower levels of HDL-cholesterol compared with average-statured women. In another cross-sectional study involving young Jamaican adults, there was a high prevalence of abdominal obesity and of low HDL-cholesterol levels.^41^

The results in this literature indicated that the consequences of stunting are complex and likely to depend on local environment, diet and developmental timing.^42^ In conclusion, the results of our study showed lower prevalence of the metabolic syndrome and the number of components presented, probably because this was a young group; however, individuals with stunting showed significantly higher prevalence of altered HDL-cholesterol and normal individuals showed significantly higher prevalence of high triglycerides and of abdominal obesity. When each metabolic syndrome component was analysed as a continuous variable, after controlling for confounding by socioeconomic and maternal conditions, stunting at age 2 years was associated with lower means of waist circumference at age 23 years, suggesting that stunting could not be considered a risk factor for metabolic syndrome components in early adulthood. Although our results were not enough to show that interventions to increase linear growth during the first 2 years of life would give some protection against the development of a risk factor for metabolic syndrome components in adulthood, the prevention of stunting is very important for the short-term consequences. In addition, other analyses could be conducted in future, taking into account the prevalence of metabolic syndrome and the association of stunting at age 2 years and altered HDL-cholesterol at age 23 years found in the present study.

## Figures and Tables

**Table 1:** Population profile of the 1982 Pelotas cohort during monitoring at 23 years of age

*Characteristics*	*Total N (%)*	*Gender*	
		*Men N (%)*	*Women N (%)*
Gender	4296 (100)	2213 (51.5)	2083 (48.5)

*Skin colour (N*=*4296)*			
White	3238 (75.4)	1658 (74.9)	1580 (75.8)
Black or mixed	908 (21.1)	471 (21.3)	437 (21.0)
Asian or indigenous	150 (3.5)	84 (3.8)	66 (3.2)

*Mother's education at birth (years) (N*=*5907)*			
0–4	1960 (33.2)	1008 (33.2)	952 (33.1)
5–8	2454 (41.5)	1288 (42.5)	1166 (40.6)
9–11	654 (11.1)	330 (10.9)	324 (11.3)
⩾12	839 (14.2)	406 (13.4)	432 (15.0)
*Family income at birth (minimum wages) (N=5885)*			
1	1288 (21.9)	666 (22.0)	622 (21.7)
1.1–3.0	2789 (47.4)	1463 (48.4)	1325 (46.4)
3.1–6.0	1091 (18.5)	544 (18.0)	547 (19.1)
>6.0–10	382 (6.5)	184 (6.1)	198 (6.9)
>10	335 (5.7)	167 (5.5)	168 (5.9)

*Mother's smoking history during pregnancy (N*=*5902)*			
Yes	2101 (35.6)	1091 (36.0)	1012 (35.2)
No	3801 (64.4)	1939 (64.0)	1861 (64.8)

*Mother's pre-gestational body mass index (kg/m*^*2*^*) (N*=*4976)*			
<18.5	388 (7.8)	211 (8.2)	177 (7.3)
18.5–24.9	3492 (70.2)	1805 (70.3)	1686 (70.0)
25.0–29.9	881 (17.7)	446 (17.4)	435 (18.1)
⩾30	215 (4.3)	104 (4.1)	111 (4.6)

*Mother's height (metres) (N*=*5808)*			
<1.52	1512 (26.0)	782 (26.3)	730 (25.8)
1.52–1.55	1475 (25.4)	737 (24.7)	737 (26.1)
1.56–1.60	1409 (24.3)	712 (23.9)	697 (24.6)
>1.60	1412 (24.3)	747 (25.1)	665 (23.5)

*Breastfeeding duration (months) (N*=*5332)*			
<1	1171 (22.0)	636 (23.3)	535 (20.6)
1–2.9	1405 (26.4)	709 (25.9)	696 (26.8)
3–5.9	1212 (22.7)	611 (22.3)	601 (23.1)
6–8,9	497 (9.3)	261 (9.5)	236 (9.1)
9–11.9	209 (3.9)	114 (4.2)	95 (3.7)
⩾12	838 (15.7)	404 (14.8)	434 (16.7)

*Weight gain during pregnancy (kg) (N*=*4408)*			
<9.6	1464 (33.2)	753 (33.0)	710 (33.4)
9.6–13.7	1446 (32.8)	737 (32.3)	709 (33.3)
⩾13.8	1498 (34.0)	791 (34.7)	707 (33.3)

*BMI at age 23 (kg/m*^*2*^*) (N*=*4288)*			
<18.5	257 (6.0)	107 (4.9)	150 (7.2)
18.5–24.9	2794 (65.2)	1424 (64.5)	1370 (65.8)
25.0–29.9	881 (20.5)	509 (23.1)	372 (17.9)
⩾30	356 (8.3)	166 (7.5)	190 (9.1)

Abbreviation: BMI, body mass index.

**Table 2:** Distribution of the prevalence of metabolic syndrome and its components according to the height-for-age *Z*-scores at 2 years of age from the 1982 Pelotas cohort study (1982–2004)

*Clinical and biochemical data*	*<−2*		*⩾−2*	
	*N*	*%*	*N*	*%*
High fasting glucose (mg/dl)	92	19.7	479	16.1
Low HDL (mg/dl)	148	30.8*	749	24.7
High triglycerides (mg/dl)	60	12.5	536	17.6*
High mean arterial pressure (mm Hg)	142	26.4	821	24.1
High waist circumference (cm)	22	4.3	218	6.6*
Overweight (wt/ht^2^)	38	5.5	339	8.0
Obesity (wt/ht^2^)	7	1.0	46	1.1

*Number of metabolic syndrome components presented*				
0	168	35.0	1247	40.7
1	200	41.7	1094	35.7
2	84	17.5	506	16.5
3	18	3.8	166	5.4
4	8	1.6	46	1.5
5	2	0.4	3	0.2
Metabolic syndrome	28	5.8	215	7.0

Abbreviation: HDL, high-density lipoprotein.

*χ*^2^-test. Asterisks indicate statistical significance. *P-value<0.05.

**Table 3:** Distribution of metabolic syndrome components according to the height-for-age *Z*-scores at 2 years of age in men and women from the 1982 Pelotas cohort study (1982–2004)

	*<−2*			*⩾−2*			
	*N*	*Mean*	*95%CI*	*N*	*Mean*	*95%CI*	*p*
*Men*							
Glycaemia (mg/dl)	265	100.2	98.4–102.2	1443	99.5	98.7–100.3	0.4785
HDL-cholesterol (mg/dl)	275	50.8	49.4–52.1	1481	51.5	50.9–52.1	0.3208
Triglycerides (mg/dl)	275	103.3	95.7–110.9	1481	117.1	113.0–121.0	0.0081*
Mean arterial pressure (mm Hg)	313	91.4	89.9–92.8	1702	91.6	91.0–92.1	0.7766
Waist circumference (cm)	312	77.3	76.2–78.3	1700	81.5	81.0–82.0	0.0000*

*Women*							
Glycaemia (mg/dl)	201	95.9	93.6–98.1	1524	94.7	94.0–95.4	0.2754
HDL-cholesterol (mg/dl)	205	55.3	53.6–57.0	1556	59.6	59.0–60.3	0.0000*
Triglycerides (mg/dl)	205	86.9	81.0–92.8	1556	97.7	94.9–100.5	0.0076*
Mean arterial pressure (mm Hg)	226	83.9	82.3–85.5	1699	84.6	84.1–85.1	0.3586
Waist circumference (cm)	204	72.8	71.4–74.2	1583	74.9	74.4–75.5	0.0055*

Abbreviations: ANOVA, analysis of variance; CI, confidence interval; HDL, high-density lipoprotein.

*T*-test and ANOVA. Asterisks indicate statistical significance. *P-value<0.05.

**Table 4:** Unadjusted and adjusted analyses of the effects of stunting at the age of 2 years on metabolic syndrome components at the age of 23 years, as stratified by gender, in the 1982 Pelotas cohort study

*Variable*	*Men*				*Women*				*Adjusted P interaction*
	*Unadjusted*		*Adjusted*		*Unadjusted*		*Adjusted*		
	*β*	*CI*	*β*	*CI*	*β*	*CI*	*β*	*CI*	
Glycaemia (mg/dl)	0.85	−1.13 to 2.83	0.92	−1.47 to 3.32	1.19	−0.87 to 3.26	1.59	−0.93 to 4.11	0.496
HDL-cholesterol (mg/dl)	−0.91	−2.39 to 0.56	0.45	−1.28 to 2.18	−4.50	−6.47 to −2.52	−1.73	−4.12 to 0.66	0.845
Triglycerides (mg/dl)	−11.90	−22.33 to −1.48	−4.74	−16.91 to 7.42	−9.61	−17.66 to −1.56	−4.01	−14.14 to 6.11	0.008
Mean arterial pressure (mm Hg)	−0.04	−1.61 to 1.53	−0.62	−2.45 to 1.22	−0.63	−2.26 to 0.99	−0.50	−2.46 to 1.46	0
Waist circumference (cm)	−4.29	−5.62 to −2.97	−2.61	−4.09 to 0.83	−1.14	−4.22 to −1.02	−2.09	−3.86 to −0.32	0

Abbreviations: CI, confidence interval; HDL, high-density lipoprotein.

Adjusted for household asset index, family income, mother's schooling, mother's smoking during pregnancy, breastfeeding duration, birth weight, mother's height, mother's pre-gestational body mass index and skin colour. Adjusted analyses were carried out using linear regression analysis. Comparisons between categories were based on tests of heterogeneity and linear trend for ordinal variables, and the lowest *P*-value was presented. Analyses were stratified according to gender, and if the *P*-value for interaction was <0.10 we considered this as evidence of interaction.
